# When annuloplasty is not enough: a case report of ventricular arrhythmias stepwise abolition after mitral valve re-repair

**DOI:** 10.1093/ehjcr/ytae305

**Published:** 2024-06-26

**Authors:** Nicolò Azzola Guicciardi, Guido Ascione, Ottavio Alfieri, Francesco Maisano, Michele De Bonis

**Affiliations:** Department of Cardiac Surgery-Valve Center—IRCCS San Raffaele Scientific Institute, Vita-Salute San Raffaele University, Via Olgettina 60, 20132 Milan, Italy; Department of Cardiac Surgery-Valve Center—IRCCS San Raffaele Scientific Institute, Vita-Salute San Raffaele University, Via Olgettina 60, 20132 Milan, Italy; Department of Cardiac Surgery-Valve Center—IRCCS San Raffaele Scientific Institute, Vita-Salute San Raffaele University, Via Olgettina 60, 20132 Milan, Italy; Department of Cardiac Surgery-Valve Center—IRCCS San Raffaele Scientific Institute, Vita-Salute San Raffaele University, Via Olgettina 60, 20132 Milan, Italy; Department of Cardiac Surgery-Valve Center—IRCCS San Raffaele Scientific Institute, Vita-Salute San Raffaele University, Via Olgettina 60, 20132 Milan, Italy

**Keywords:** Mitral valve prolapse, Mitral valve repair, Arrhythmic mitral valve prolapse, Ventricular arrhythmias, Case report

## Abstract

**Background:**

Some patients affected by mitral valve (MV) prolapse (MVP) are at higher risk of ventricular arrhythmias (VAs), but the underlying pathogenesis, as well as the effects of surgery on VA, remain not fully understood. Mitral valve repair, however, represents a privileged point of view to deepen the understanding of arrhythmogenesis in this context. Hence, we report an interesting case of MV re-repair.

**Case summary:**

A 52-year-old man was referred to our institution for severe mitral regurgitation (MR) due to P2 prolapse in the context of myxomatous MV degeneration. Pre-operative imaging showed systolic mitral annular disjunction, left ventricular (LV) wall curling, Pickelhaube’s sign, and a prolapsing tricuspid valve (TV) with only mild regurgitation. Twenty-four-hour electrocardiogram (ECG) Holter revealed a significant burden of premature ventricular contractions (PVCs), most of them originating from anterior papillary muscle (APM), posterior papillary muscle (PPM), and mitral annulus (MA). Quadrangular resection of P2 and mitral annuloplasty were performed. One year later, relapse of severe MR due to a residual P2M1 prolapse occurred. Twenty-four-hour ECG Holter showed no PVCs from PPM and MA, while those from APM persisted. A central edge-to-edge repair was effectively used to fix the residual prolapse. After 1 year from REDO surgery, a third ECG Holter confirmed the absence of any remaining LV PVCs, but still few ectopic beats originating from TV were recorded.

**Discussion:**

Here, we report a case of VA resolution after specific, anatomical triggers addressing surgical gestures. Our experience confirms that MV surgery may have a role in MVP patients’ arrhythmias correction.

**Learning points**

- Arrhythmic profile of patients with mitral valve prolapse undergoing surgery should be evaluated through a multimodal approach (continuous rhythm monitoring, trans-oesophageal echocardiography, and myocardial assessment to characterize fibrosis extension), in order to provide an exhaustive risk stratification.
- Addressing anatomical triggers of ventricular ectopic beats through specific surgical manoeuvres may abolish arrhythmias in patients in which arrhythmogenesis is mainly due to prolapse-induced stretch of left ventricular myocardium (i.e. before fibrosis develops).

## Introduction

Mitral valve (MV) prolapse (MVP) is common among general population, with a prevalence of around 2–3%.^1^ A subgroup of patients with MVP is known to be at higher risk of malignant ventricular arrhythmias (VAs) and sudden cardiac death (SCD).^2^ Despite an increasing interest in the topic during the last few years, many aspects, including risk stratification, therapeutic management, and genetic background of this condition,^3^ remain still very controversial.

Recently, the European Heart Rhythm Association consensus summarized the latest evidence in this field, providing a common strategy to recognize and treat patients with MVP experiencing VA.^4^ Namely, a proper arrhythmic MVP phenotype has been depicted: subjects more prone to show VA are usually affected by Barlow’s disease, with bileaflet prolapse, mitral annular disjunction (MAD), systolic left ventricular (LV) wall curling, Pickelhaube’s sign, fibrosis of papillary muscles (PMs), and infero-basal LV wall.^5^ Moreover, MVP patients’ risk of SCD has been stratified according to the severity of VA [premature ventricular contractions (PVCs), non-sustained ventricular tachycardias (VTs), polymorphic VT, and ventricular fibrillation].^5^

Whether mitral surgery has a role in arrhythmias abolition in these subjects is still a matter of debate.

In our preliminary experience, we showed that approximately one-third of patients with Barlow’s disease undergoing MV repair have a significant pre-operative VA burden and that in more than half of them, this burden disappears after surgery.^6^ Notably, most of the arrhythmogenic patients (68%) at baseline experienced PVCs originating from posterior PM (PPM), while PVCs from mitral annulus (MA) were present in only 43% of the cases. Although many reports gave to MAD a central role in MVP arrhythmogenesis,^4^ it may not be the only involved actor. Hence, besides annuloplasty, providing correction of MAD and systolic curling, prolapse-addressing manoeuvres potentially play a pivotal role in arrhythmias treatment.

More data are needed to corroborate these hypotheses, but surgical experience in this field could help in deepening the understanding of MVP arrhythmogenesis. Therefore, we describe an interesting case of MV re-repair.

## Summary figure

A 52-year-old man with mitral P2 prolapse and myxomatous degeneration determining severe MR was admitted to our institution for MV surgery. Pre-operative trans-oesophageal echocardiogram (TOE) revealed systolic MAD and systolic LV infero-basal wall curling. Tricuspid valve (TV) was affected by prolapse determining mild regurgitation. Twenty-four-hour electrocardiogram (ECG) Holter monitoring revealed a significant burden of PVCs (>5% in 24 h), most of them originating from anterior PM (APM), PPM, and MA (*Figure frame A*). Quadrangular resection of P2 and annuloplasty with a 37 mm posterior band were performed. After 1 year, the patient experienced relapse of severe MR due to a residual P2M1 prolapse. Twenty-four-hour ECG Holter was repeated. Premature ventricular contractions from PPM and MA disappeared, while those from APM persisted (*Figure frame B*). A central edge-to-edge repair was employed to fix the residual prolapse. After 1 year from REDO surgery, a third ECG Holter confirmed the absence of any remaining LV PVCs, but still few ectopic beats originating from TV were recorded (*Figure frame C*).

**Figure:**
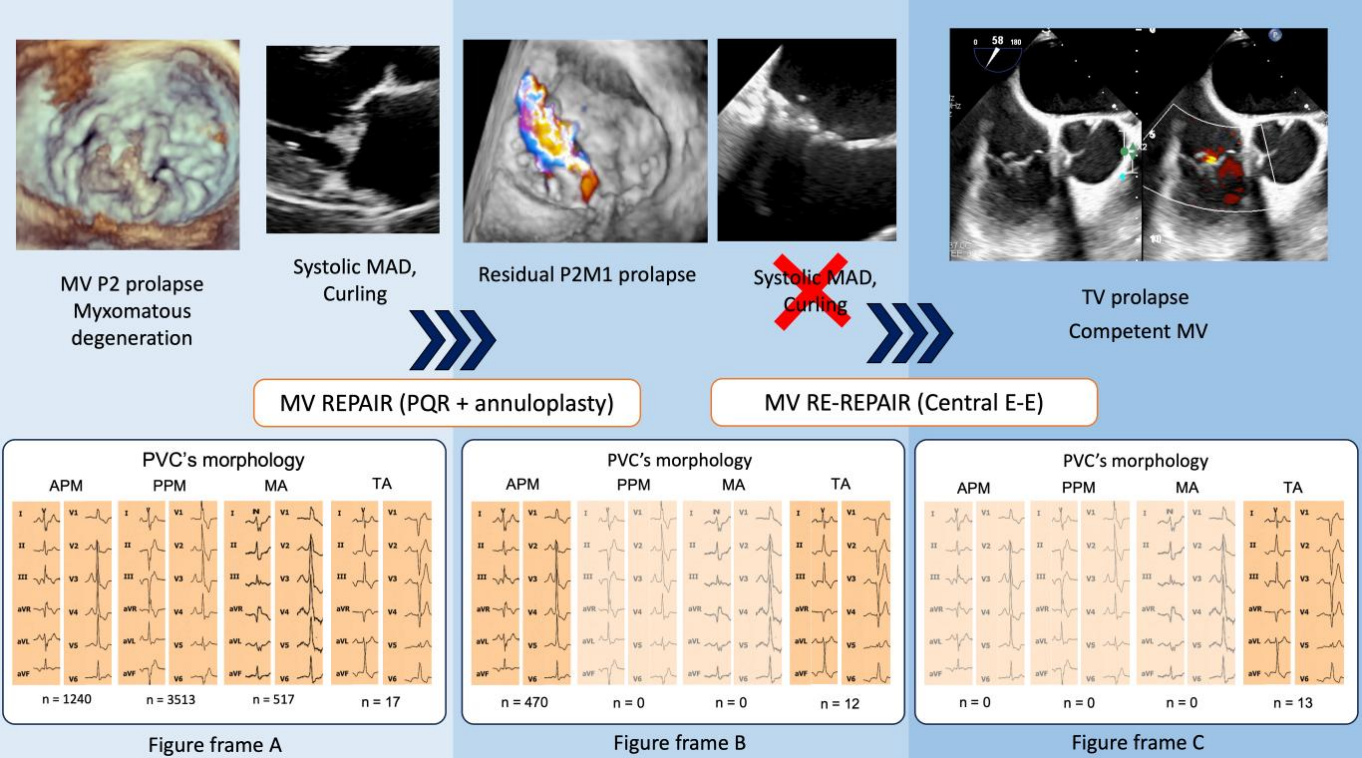


## Case presentation

**Figure 1:**
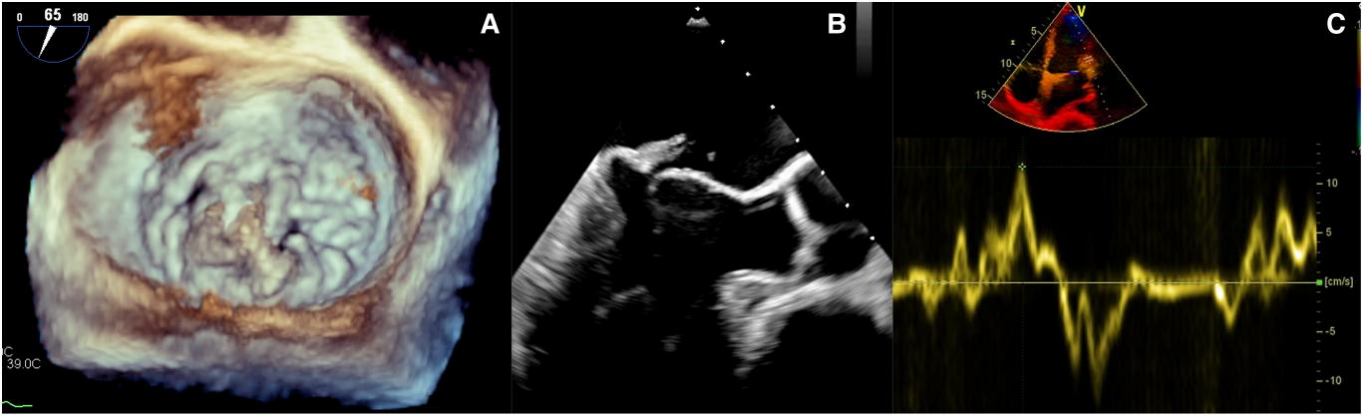
(*A*) Three-dimensional (3D) trans-oesophageal echocardiogram, surgical view. Mitral posterior leaflet’s central scallop (P2) prolapse due to myxomatous degeneration. (*B*) Two-dimensional (2D) trans-oesophageal echocardiogram, three-chamber view. Systolic mitral annular disjunction and curling of left ventricular infero-basal wall. (*C*) Pickelhaube’s sign.

**Figure 2:**
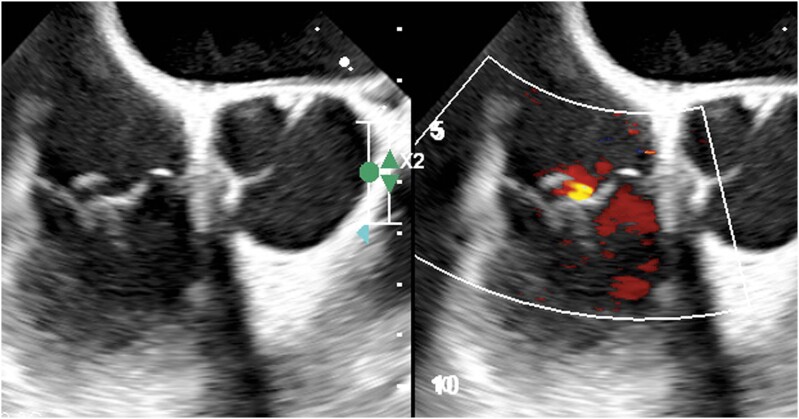
Two-dimensional (2D) trans-oesophageal echocardiogram, right ventricular outflow tract view. Tricuspid valve leaflets appear redundant and prolapsing with associated mild regurgitation.

A 52-year-old man was referred to our institution for severe symptomatic mitral regurgitation (MR). Pre-operative TOE showed MVP due to myxomatous degeneration involving both leaflets. The main lesion was prolapse of the central scallop of the posterior leaflet (P2) (*Figure 1A*). Systolic MAD (8 mm), LV infero-basal wall curling, and Pickelhaube’s sign were present (*Figure 1B* and *C*). Of note, also TV was affected by diffuse leaflets prolapse resulting in mild regurgitation (*Figure 2*). The pre-operative coronary angiography did not show any coronary stenosis. At pre-operative 24-hour electrocardiogram (ECG) Holter, 5167 PVCs (>5%/day) were detected. The most common PVC morphology was PPM (62%), followed by APM (24%) and posterior MV annulus (10%). Premature ventricular contraction details are shown in the Summary figure. The patient was scheduled for minimally invasive elective MV repair and, given the prevalent posterior leaflet prolapse, underwent P2 quadrangular resection and concomitant annuloplasty with a posterior band (37 mm). At discharge, a mild to moderate MR persisted.

**Figure 3:**
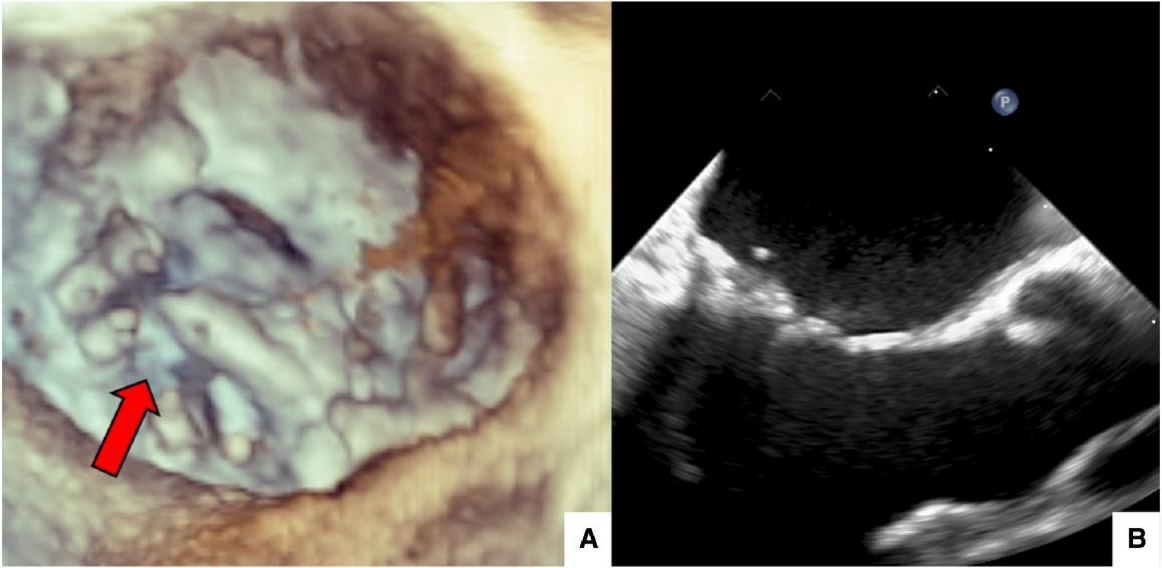
(*A*) Three-dimensional (3D) trans-oesophageal echocardiogram, surgical view. While the medial portion of posterior leaflet’s central scallop (P2M2) is fixed, residual prolapse of its lateral portion (P2M1) persists. (*B*) Two-dimensional (2D) trans-oesophageal echocardiogram, three-chamber view. Systolic mitral annular disjunction and left ventricular infero-basal wall curling have been effectively abolished by annuloplasty.

One year after, the patient presented with recurrence of severe MR, in the context of residual prolapse of the lateral portion of P2 (P2M1) (*Figure 3A*). He was consequently scheduled for REDO-MV surgery. Pre-REDO 24-hour ECG Holter revealed 517 PVCs, of which 91% originating from APM. Neither VAs from PPM nor from MV annulus were anymore detected. Pre-operative cardiac computed tomography scan with myocardial characterization did not point out any spot of LV fibrosis. Mitral re-repair was subsequently performed: edge-to-edge technique was effectively used to fix the residual P2M1 prolapse. At 1-year follow-up, a trans-thoracic echocardiography showed trivial residual MR and mild tricuspid regurgitation. Interestingly, a third 24-hour ECG Holter revealed only 13 PVCs, all originating from tricuspid annulus. The tricuspid annular PVC burden remained stable across the different rhythm evaluations (17, 12, and 13 PVCs respectively recorded in the first, second, and third 24-hour ECG Holter). No antiarrhythmic therapy was ever introduced.

## Discussion

The mechanisms underlying MVP patients’ VAs remain not fully understood. One of the most shared hypotheses is that arrhythmias could be generated by prolapse-induced PM and infero-basal LV wall mechanical stress during late systole. Later on, once repeated stress has evolved in fibrosis, fibrosis-related re-entry circuits and their interaction with stretch-activated depolarizations may be the main responsible of ventricular ectopic beats.^7,8^

This case offers interesting hints about arrhythmogenesis in MVP patients. Indeed, we had the opportunity to document step by step how specific surgical gestures may affect VA in this context.

After the first MV repair, systolic MAD and curling were fixed (*Figure 3B*) and, consequently, PVCs from MA disappeared. Premature ventricular contractions originating from PPM vanished as well, consistently with P2M2 prolapse abolition, while those from APM persisted. The persistence of mechanical traction on the tip of APM, in the context of residual P2M1 prolapse, was most likely responsible for this failure to eradicate APM ectopic beats. In fact, once P2M1 prolapse was corrected during REDO surgery, VAs originating from APM were not recorded anymore. Interestingly, TV was not treated and the number of PVCs originating from its annulus remained stable during the observation period. The prognostic implications of these tricuspid ectopic beats are poorly known, but myxomatous degeneration of TV has already been described and linked with an increased arrhythmic risk.^9^

Our experience suggests that the pathogenesis of VAs in MVP patients has two distinct stages: first, prolapse-induced traction on PMs and curling of the infero-basal LV wall produce VAs that can be abolished if the triggers are addressed surgically in a timely fashion.^10^ Later on, repeated mechanical stimulation would eventually lead to inveterate fibrosis, and at that point, MV repair may not have an antiarrhythmic effect anymore.^11^ Further comprehensive analyses (with both myocardial characterization and electrophysiological studies) are needed to better characterize the arrhythmic substrate of MVP patients.

## Lead author biography


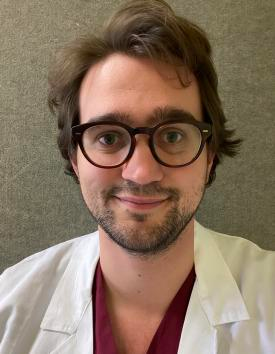


Dr Nicolò Azzola Guicciardi graduated from ‘Vita-Salute’ S. Raffaele University, Milan, and is currently attending his residency at the same centre, in the Department of Cardiac Surgery. Together with Dr Guido Ascione, who is the shared lead author, he is committed to better understand arrhythmic mitral valve prolapse and its relationship with mitral valve surgery. His interests include valvular heart diseases and their treatment, either surgical or transcatheter.

## Data Availability

All data are incorporated into the article and its online supplementary material.
